# Supplementary material for: Experience of “One Stop TB Diagnostic Solution” Model in Engaging a Private Laboratory for End-to-End Diagnostic Services in the National TB Elimination Program in Hisar, India
Source: Diagnostics (Basel). 2023 Aug 31;13(17):2823. doi: 10.3390/diagnostics13172823 (PMC10486804; doi:10.3390/diagnostics13172823)

Supplementary Figure S1. F1 The sample collection route map covered by Runners along with CXR facilities.

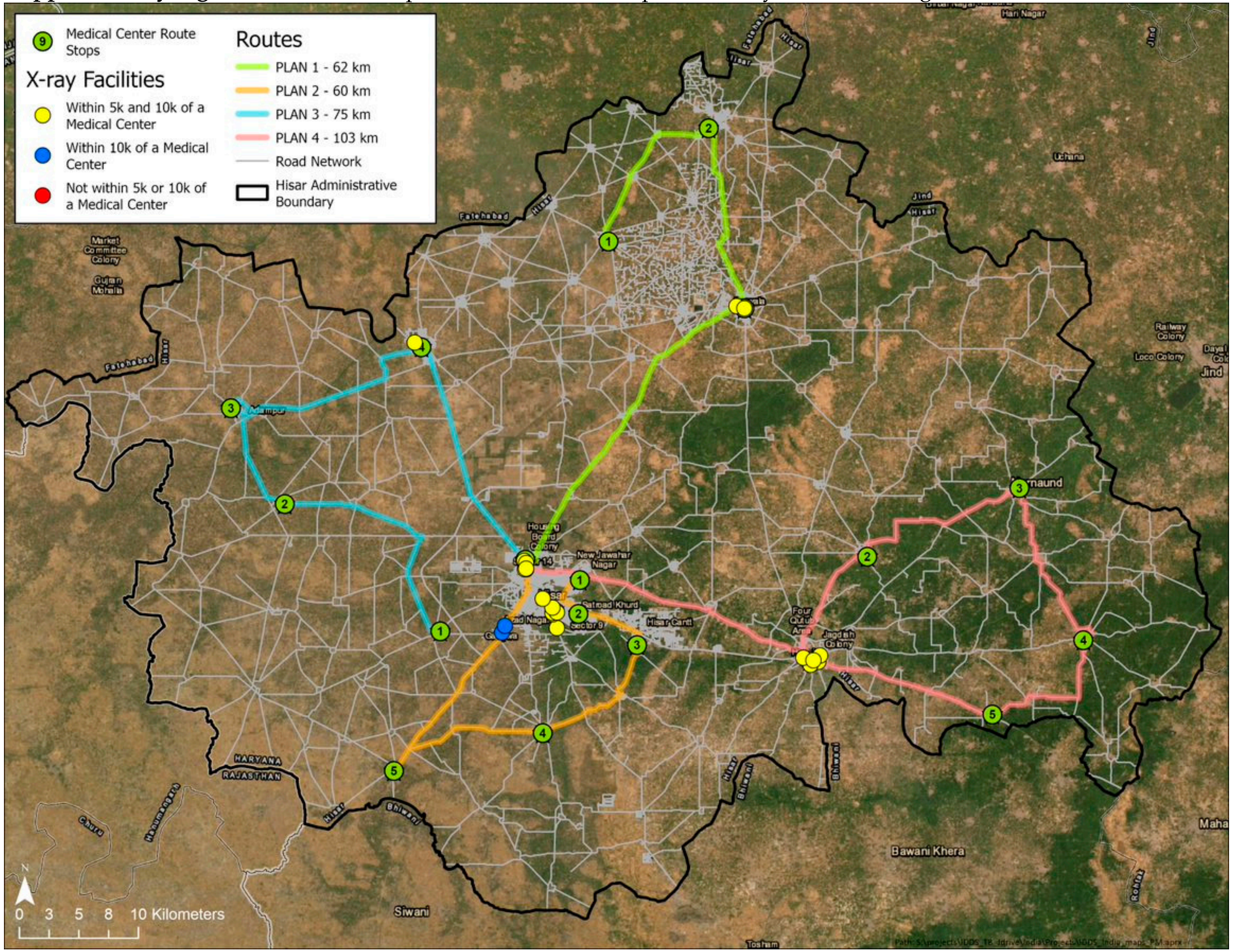

Supplement: Supplementary file 1 [file diagnostics-13-02823-s001.zip › Supplementary Figure S1.pdf]
