# Supplementary material for: Experience of “One Stop TB Diagnostic Solution” Model in Engaging a Private Laboratory for End-to-End Diagnostic Services in the National TB Elimination Program in Hisar, India
Source: Diagnostics (Basel). 2023 Aug 31;13(17):2823. doi: 10.3390/diagnostics13172823 (PMC10486804; doi:10.3390/diagnostics13172823)
Supplement: Supplementary file 1 [file diagnostics-13-02823-s001.zip › Supplementary Table S1.pdf]

Supplementary Table S1. Comparative analysis of completing diagnostic algorithm for Rifampicin Sensitive Tuberculosis

	Rifampicin Sensitive (RS) TB Diagnostic Algorithm	Uma Shankar S, et al 2021(Year 2018) [12]		One-Stop TB Model (Year 2022)	
**sl no**	**Particulars**	**Number**	**Percentage**	**Number**	**Percentage**
1	Number of RS TB Patients diagnosed at Xpert Lab	1,590		1,996	
2	Number of samples reached the reference lab	1,170	74%	1,996	100%
3	FL-LPA not done	39*	3%	0	
4	FL-LPA not done	1131	97%	1,806**	90%
i	H and R Sensitive	1,056	93%	1,692	94%
ii	H Resistant and R Sensitive	51	5%	108	6%
iii	R Resistant only	3	1%	1	<1%
Iv	H and R Resistant			5	<1%
iv	No results	21	2%	0	
5	SLLPA done	11	20%	114	100%
6	SL-LPA not done	43	80%	0	0
i	FQ and SLI Sensitive	7	63%	99	87%
ii	FQ and/ or SLI Resistant	0		15	13%
iii	No results	4		0	0
7	Culture done	43	40%	114	100%
8	Result for LC DST (Moxi, PZA,Lzd)			Moxi (3/108,) Lzd (1/108), PZA (14/108).	
9	Number of RS Patients completing the diagnostic algorithm	1,106	95%	1985#	99%

**Reasons not listed ** smear negative & culture-negative specimens excluded #11 insufficient samples did not complete sequential tests. Moxi – moxifloxacin, Lzd- Linezolid, PZA – pyrazinamide, H – Isoniazid, R – Rifampicin, FL-LPA – First Line LPA, SL-LPA – Second Line LPA, FQ – Fluroquinolone. RS – Rifampicin Sensitive. SLI – Second Line Injectable. LC – DST – Liquid Culture Drug susceptibility test.*
