# Supplementary material for: Experience of “One Stop TB Diagnostic Solution” Model in Engaging a Private Laboratory for End-to-End Diagnostic Services in the National TB Elimination Program in Hisar, India
Source: Diagnostics (Basel). 2023 Aug 31;13(17):2823. doi: 10.3390/diagnostics13172823 (PMC10486804; doi:10.3390/diagnostics13172823)
Supplement: Supplementary file 1 [file diagnostics-13-02823-s001.zip › Supplementary Table S2.pdf]

Supplementary Table S2 - Comparative analysis of completing diagnostic algorithm for Rifampicin Resistant Tuberculosis

	RR TB Diagnostic Algorithm	Uma Shankar S, et al 2021(Year 2018) [12]		One-Stop TB Model (Year 2022)	
**sl no**	**Particulars**	**Number**	**Percentage**	**Number**	**Percentage**
1	Number of RR-TB patients diagnosed at Xpert Lab	64		134	
2	Number whose samples reached reference lab	35	55%	134	
i	FL-LPA done	n.a		111	100%
ii	FL-LPA not done	n.a		2	1%
iii	H and R Sensitive	n.a		4	3%
iv	R Resistant	n.a		26	23%
v	H and R Resistant	n.a		81	73%
3	SL-LPA done	14	40%	107	96%
4	SL-LPA Not done	21	60%	2	2%
i	FQ and SLI sensitive	9	64%	81	76%
ii	FQ and SLI Resistant	2	15%	26	24%
iii	No results available	3	21%	0	0
5	Culture done	8	31%	107	100%
i	Result for LC-DST (Moxi, PZA, Lzd)			Moxi (8/104)<br>Lzd (1/104)<br>PZA (26/104).	
6	Number of RR-TB patients completing the diagnostic algorithm	17	49%	132	98%

Moxi – moxifloxacin, Lzd- Linezolid, PZA – pyrazinamide, H – Isoniazid, R – Rifampicin, FL LPA – First Line LPA, SL LPA – Second Line LPA, FQ – Fluroquinolone. RS – Rifampicin Sensitive. SLI – Second Line Injectable. LC – DST – Liquid Culture Drug susceptibility test.
