# Supplementary material for: Experience of “One Stop TB Diagnostic Solution” Model in Engaging a Private Laboratory for End-to-End Diagnostic Services in the National TB Elimination Program in Hisar, India
Source: Diagnostics (Basel). 2023 Aug 31;13(17):2823. doi: 10.3390/diagnostics13172823 (PMC10486804; doi:10.3390/diagnostics13172823)
Supplement: Supplementary file 1 [file diagnostics-13-02823-s001.zip › Supplementary Table S3.pdf]

Supplementary Table S3. Comparative analysis for Turnaround Time as proposed in NTEP

One-Stop TB Model (Year 2022)*	NTEP suggested TAT (in days)			Uma Shankar S. et al 2021 [12] * (in days)	One-Stop TB Model (Year 2022)* (in days)		
Test Name	Pre-Lab TAT	Lab TAT	Total lab TAT	Total TAT	Pre-Lab TAT	Lab TAT	Total TAT
Xpert test	1-2	1-2	2- 4	1 (1–2)	1	1	1 (1-1)
FL-LPA	1-3	2-3	3-6	5 (4–6)	n.a	4 (3-5)	4 (3-5)
SL – LPA				16(8-30)	n.a	2.5 (1-4)	2.5 (1-4)
LC DST	n.a	5-7 (LPA) 22-48 (LC DST) (in most cases 30 days)	27-55	n.a	n.a	22 (17-32)	22 (17-32)

*n.a - not available * median and interquartile range (IQR)*
