# Supplementary material for: Experience of “One Stop TB Diagnostic Solution” Model in Engaging a Private Laboratory for End-to-End Diagnostic Services in the National TB Elimination Program in Hisar, India
Source: Diagnostics (Basel). 2023 Aug 31;13(17):2823. doi: 10.3390/diagnostics13172823 (PMC10486804; doi:10.3390/diagnostics13172823)
Supplement: Supplementary file 1 [file diagnostics-13-02823-s001.zip › Supplementary Table S4.pdf]

**Supplementary Table S4.** Rifampicin Indeterminant Specimens completing Diagnostic Algorithm

| | **Rifampicin Indeterminant TB Patients** | **One-Stop TB Model** | |
|---|---|---|---|
| sl no | Particulars | Number | Percentage |
| 1 | Number of RI-TB patients diagnosed at Xpert Lab | 22 | |
| 2 | Number whose samples reached the reference lab | 22 | |
| i | FL-LPA done | 16 | 100% |
| ii | H and R Sensitive | 10 | 63% |
| iii | R Resistant | 0 | 0% |
| iv | H and R Resistant | 6 | 37% |
| 3 | FL-LPA not done | 1 | 5% |
| 4 | Liquid Culture done | 22 | 100% |
| | Liquid Culture Positive (included into FL-LPA tests) | 16 | 73% |
| 5 | SL- LPA done | 16 | 100% |
| i | FQ and SLI sensitive | 8 | 77% |
| ii | FQ and/ or SLI Resistant | 2 | 23% |
| iii | No results available | 0 | 0 |
| 6 | SL LPA Not done | 0 | 0% |
| 7 | Liquid Culture DST done | 16 | 100% |
| i | Result for LC DST (Moxi, PZA, Lzd) | Moxi (0/15)<br>Lzd (0/15)<br>PZA (1/15). | |
| 8 | Number of Rifampicin Resistant patients completing the diagnostic algorithm | 21 | 95% |

*Moxi – moxifloxacin, Lzd- Linezolid, PZA – pyrazinamide, H – Isoniazid, R – Rifampicin, FL LPA – First Line LPA, SL LPA – Second Line LPA, FQ – Fluroquinolone. RS – Rifampicin Sensitive. SLI – Second Line Injectable. LC – DST – Liquid Culture Drug susceptibility test.*
